# Clinico-pathological correlation of lacrimal caruncle tumors: a retrospective analysis over 22 years at the University Eye Hospital Bonn

**DOI:** 10.1007/s00417-021-05464-x

**Published:** 2021-10-28

**Authors:** A. C. Clemens, K. U. Loeffler, F. G. Holz, M. C. Herwig-Carl

**Affiliations:** 1 Department of Ophthalmology, University Hospital Bonn, Ernst-Abbe-Str. 2, 53127 Bonn, Germany; 2 Division of Ophthalmic Pathology, Department of Ophthalmology, University Hospital Bonn, Bonn, Germany; 3 Center for Integrated Oncology Aachen Bonn Cologne Dusseldorf (CIO ABCD), Bonn, Germany

**Keywords:** Caruncle, Tumor, Clinic-pathologic correlation, Oncocytoma, Nevus, Sebaceous gland hyperplasia

## Abstract

**Purpose:**

The lacrimal caruncle is composed of numerous structures including different glands as well as hair follicles. Accordingly, the spectrum of benign and malignant lesions is broad, and the clinical diagnosis is often challenging. Here we systematically analyzed excised caruncular tumors over the past 22 years with special emphasis on the clinico-pathological correlation to provide a guidance for clinicians.

**Methods:**

Retrospective evaluation with clinico-pathologic correlation of surgically removed caruncular tumors between 1998 and 2020 at a tertiary referral center.

**Results:**

Eighty-two caruncular tumors were identified in the respective period. The patients were between 11 and 85 years of age (mean, 46.8 years; median, 49 years). Nevi (*n* = 35), cystic lesions (*n* = 14), oncocytoma (*n* = 9), papilloma (*n* = 8), sebaceous gland hyperplasia (*n* = 8), and reactive lymphoid hyperplasia (*n* = 4) were observed most frequently. Besides, we are the first reporting herniated orbital fat accompanied by a pyogenic granuloma. 2.4% (*n* = 2) were malignant tumors (sebaceous gland carcinoma, conjunctival intraepithelial neoplasia with pyogenic granuloma).

**Conclusion:**

Caruncular tumors show a broad spectrum of mostly benign tumors. They can occur in patients of any age. However, 8/9 oncocytomas and both malignant lesions were detected in patients older than 60 years. Although the clinical diagnosis was confirmed in only 68.3% by the histopathological analysis, the two malignant lesions were identified as such already clinically. Caruncular lesions with a history of growth or other signs of malignancy should be excised followed by detailed histopathological examination to allow a final diagnosis and exclude rare malignant tumors with lethal potential. 
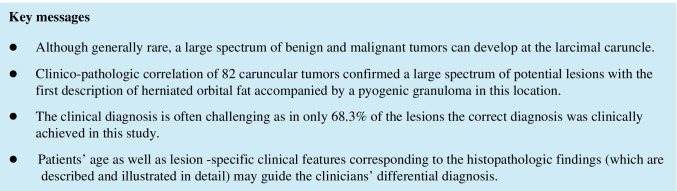

**Supplementary Information:**

The online version contains supplementary material available at 10.1007/s00417-021-05464-x.

## Introduction

The lacrimal caruncle (herein referred to as caruncle) is located in the medial angle of the eyelids. It is covered partly by non-keratinized squamous epithelium with numerous goblet cells and partly by keratinized stratified squamous epithelium. The caruncle is composed of loose connective tissue rich in fibroblasts and may also contain adipose tissue. It contains numerous skin appendages such as hair follicles, sebaceous and sweat glands as well as occasionally accessory lacrimal glands (accessory lacrimal glands of Popoff), and striated muscle fibers. Melanocytes are present in the basal layer of the surface epithelium. The composition of the caruncle with its various structures is responsible for the variety of benign and malignant tumors arising in this specific location.

Caruncular tumors are rare compared to conjunctival lesions; however, most of them (95%) are benign [1–5]. The incidence of caruncular lesions in the literature ranges from 0.3 to 1.1% [1–3, 6, 7]. There are only some other reports on larger cohorts of caruncular tumors with our series being one of the largest one [1–11] [three larger, older studies are summarized in [6]].

In this study, we report 82 lesions surgically excised during the last 22 years at the University Eye Hospital Bonn, Germany. This clinico-pathological correlation aims to provide guidance for clinicians with regard to differential diagnoses and treatment of patients with caruncular lesions.

## Material and methods

All primary caruncle tumors were surgically excised between 1998 and 2020 at the University Eye Hospital Bonn and histopathologically diagnosed at our Ophthalmopathologic Laboratory. The suspected clinical and final histopathological diagnoses were correlated. For this study, the clinical data including the clinical diagnosis of the patients was retrospectively evaluated. The histological evaluation was performed by light microscopy using routine hematoxylin–eosin staining (HE) and periodic acid-Schiff reaction (PAS) as well as immunohistochemical stains where necessary (e.g., for nevus and reactive lymphoid hyperplasia, RLH). Caruncular tissue which was excised during pterygium surgery was not included in the study nor was conjunctival intraepithelial neoplasia of the tarsal/bulbar conjunctiva involving the caruncle.

The number and histological diagnosis of surgically removed conjunctival lesions which were excised during the same period were recorded allowing for a comparison with regard to frequency of conjunctival and caruncle tumors.

The research was conducted in adherence to tenets of the Declaration of Helsinki. Ethics Board Approval of the University of Bonn was granted (328/16).

## Results

Between 1998 and 2020, 82 excised caruncular tumors were identified. The patients were between 11 and 85 years of age (mean, 46.8 years; median, 49 years) and more frequently female (60%, Table 1). The right eye was affected in 37 cases (55%). Most of the patients reported slow growth of the caruncular lesion over time. The diameter of the lesions ranged from 1 × 1 × 0.5 mm (sweat gland cyst) to 11 × 10 × 8 mm (reactive lymphoid hyperplasia, RLH).

**Table.1:** Epidemiological and clinical characteristics of the caruncle tumors

Diagnosis	Number (%)	Age (mean/median, in years) [age range in yrs]	Gender (M:F)	Correct clinical diagnosis (%)
**Benign lesions**	**80 (97.6)**	**46.3/46.5**	**32:48**	**54 (67.5)**
**Benign melanocytic lesions**	35 (42.7)	37.8/33 [11–81]	5:30	25 (71.4)
Pigmented	25	30.8	3:22	22 (88)
Amelanotic	10	55.2	2:10	3 (30.0)
**Cystic lesions**	14 (17.1)	43.1/36 [22–76]	7:7	9 (64.3)
Keratin cyst	7	29.6/29	3:4	5 (71.4)
Sweat gland cyst	5	54.6/60	3:2	3 (60)
Conjunctival implantation cyst	1	76	0:1	1 (100)
Resorptive cyst	1	48	1:0	0
**Oncocytoma**	9 (11)	71.9/70 [55–85]	5:4	5 (55.5)
**Papilloma**	8 (9.8)	46.3/45 [27–66]	5:3	8 (100)
**Sebaceous gland hyperplasia**	7 (8.5)	61/58 [37–83]	7:0	6 (85.7)
**Reactive lymphoid hyperplasia**	4 (4.9)	35/28.5 [21–62]	2:2	1 (25.0)
**Others**	3 (3.7)	65.3/57 [55–84]	1:2	0 (0.0)
Regular caruncle	1	55/55	1:0	0 (0.0)
Dermoid	1	57/57	0:1	0 (0.0)
Herniated orbital fat (+ pyogenic granuloma)	1	84/84	0:1	0 (0.0)
**Malignant lesions**	**2 (2.4)**	**66/66 [60–72]**	**1:1**	**2 (100)**
**CIN**	1 (1.2)	60/60	0:1	1 (100.0)
**Carcinoma**	1 (1.2)	72/72	1:0	1 (100.0)
**Σ**	**82 (100)**	**46.8/49 [11–85]**	**33:49**	**56 (68.3)**

Legend: *F* female; *M* male; *yrs* years

Benign, malignant and all lesions and resp. numbers/age/gender/diagnosis are in boldface as well as main diagnosis terms

All lesions were unilateral and solitary. The most frequent tumors were benign melanocytic lesions (*n* = 35) and papillomas (of the conjunctival epithelium, *n* = 14), followed by oncocytomas (*n* = 9), cystoid lesions (*n* = 9), and sebaceous gland hyperplasia (*n* = 7). Only 2.4% patients of the cohort (*n* = 2) exhibited a malignancy (carcinoma, pyogenic granuloma with conjunctival intraepithelial neoplasia (CIN), Table 1). While papillomas, sebaceous gland hyperplasia, melanocytic lesions (in particular the pigmented ones), and cysts were mostly diagnosed already clinically, the suspected clinical diagnosis for the other lesions frequently differed from the final histological diagnosis. However, both malignant lesions were identified as such by their clinical appearance and growth characteristics which are described in detail below. In total, 68% (*n* = 56) of the lesions were correctly diagnosed clinically respectively the final histological diagnosis was already included in the tentative clinical diagnosis.

The intra- and postoperative course was free of complications except for the malignant lesions: The patient with epithelial carcinoma died several months after the initial diagnosis. The patient with conjunctival intraepithelial neoplasia (CIN) underwent complete surgical excision followed by two cycles of mitomycin C 0.04%. After 2 years, the patient developed a recurrence which was again treated with surgical excision and another cycle of mitomycin C 0.04%. The patient is now free of recurrence for 4 years [12].

Compared to the conjunctival tumors excised during the same period (*n* = 1183) at our institution, caruncular tumors (*n* = 82) are much rarer and account for only 6.4% of the surgically removed lesions of the anterior segment.

Oncocytoma and sebaceous gland hyperplasia are — although rarely observed at other ocular locations — typical tumors of the caruncle. The other lesions frequently occur at other sites of the ocular adnexae. The age spectrum between the tumors differed with oncocytoma and malignant lesions occurring in older patients, typically around the age or retirement, while nevi, cystic lesions, and RLH were more often diagnosed at a younger age (supplemental Fig. 1).

The lesions were further analyzed in detail with special emphasis on the clinico-pathological correlation:

### Benign lesions

#### Benign melanocytic lesions (42.7%, *n* = 35; Fig. 1)

**Fig. 1:**
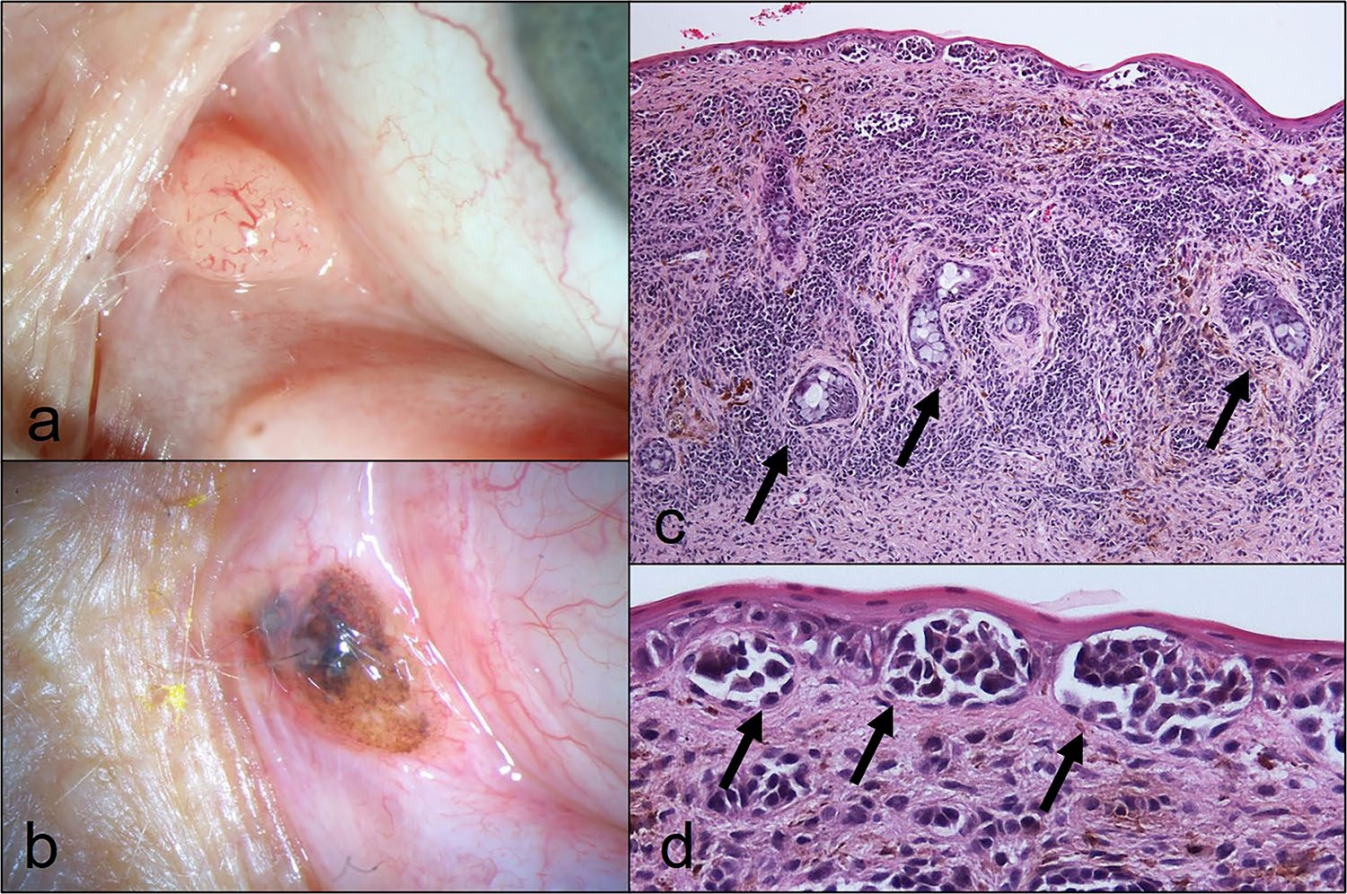
Amelanotic nevus. Clinical picture of an amelanotic caruncular nevus (**a**). Clinical picture of an amelanotic caruncular nevus with tiny, visible cysts (**b**). Pigmented nevus. Histologic figure of a caruncular nevus with several conjunctival inclusion cysts (arrows). The melanocytes are arranged in nests (**c**; H&E stain, 40 ×). Higher magnification of junctional activity (arrows, d; H&E stain, 200 ×)

Clinical findings: brownish or whitish-reddish (amelanotic lesions); papillomatous (combined lesion of nevus and papilloma, *n* = 2).

Histology: stromal lesion composed of nest of melanocytes, many of them showing pseudonuclear inclusions; occasionally junctional activity (JA).

JA occurred more frequently in pigmented lesions compared to amelanotic melanocytic lesions (supplemental table). Patients with JA were typically younger. However, focal JA was detected in patients up to 57 years without any sign of malignant transformation.

#### Cystic lesions (17.1%, *n* = 14; supplemental Fig. 2)

Clinical findings: yellowish (keratin cyst), variable (for other types).

Histology: cyst lined by keratinized squamous epithelium (keratin cyst)/non-keratinized epithelium with apocrine/eccrine secretion (sweat gland cyst)/non-keratinized squamous epithelium with goblet cells (conjunctival implantation cyst)/no epithelium (resorptive cyst); lumen filled with keratin lamellae (keratin cyst) or eosinophilic material.

#### Oncocytoma (11%, *n* = 9; syn. oxyphilic adenoma; Fig. 2)

**Fig. 2:**
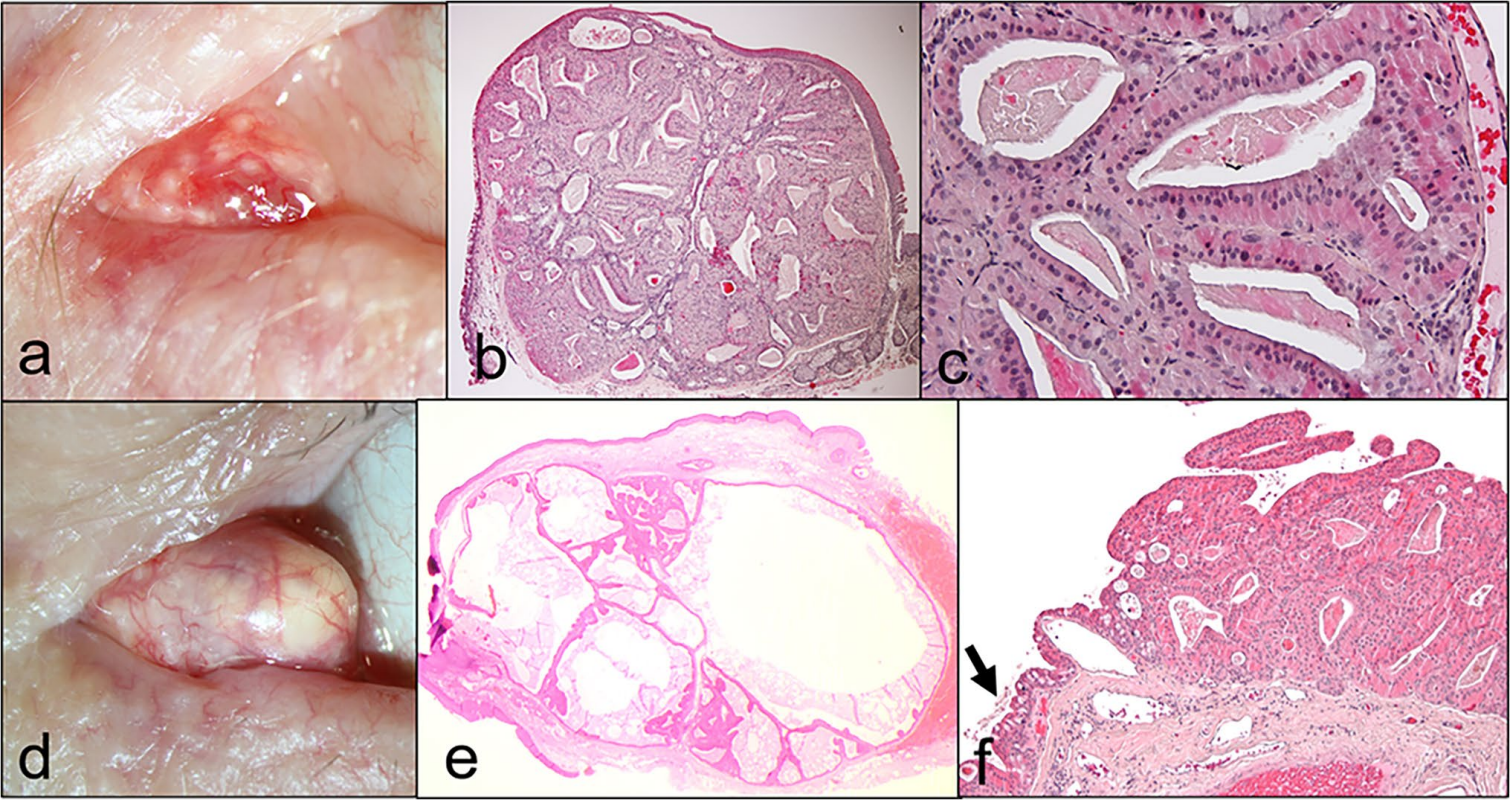
Oncocytoma. Clinical picture of a reddish lesion of the caruncle (**a**). The corresponding histologic figure shows a compact lesion with cystic spaces (**b**; H&E stain, 40 ×). Higher magnification shows a columnar, granular, eosinophilic epithelial lining with myoepithelial cells on its base (**c**; H&E stain, 100 ×). Clinical picture of a whitish to yellowish cystic lesion at the caruncle (**d**). The corresponding histologic figure shows a multilobulated cyst (**e**; H&E stain, ×). There are goblet cells (arrow) present intermingled with the regular oncocytomatous epithelial lining (**f**; H&E stain, 100 ×)

Clinical findings: reddish or whitish, with a smooth surface and a nodular or cystoid appearance.

Histology: cystic cavities lined by a proliferating epithelium; cells with eosinophilic and granular cytoplasm (due to abnormal mitochondriae, detected by TEM), accompanying inflammatory component possible.

*The histologic appearance of oncocytoma resembles an apocrine epithelium — however, (accessory) lacrimal glands exhibit an eccrine secretion.*

#### Papilloma (9.8%, *n* = 8; Fig. 3)

**Fig. 3:**
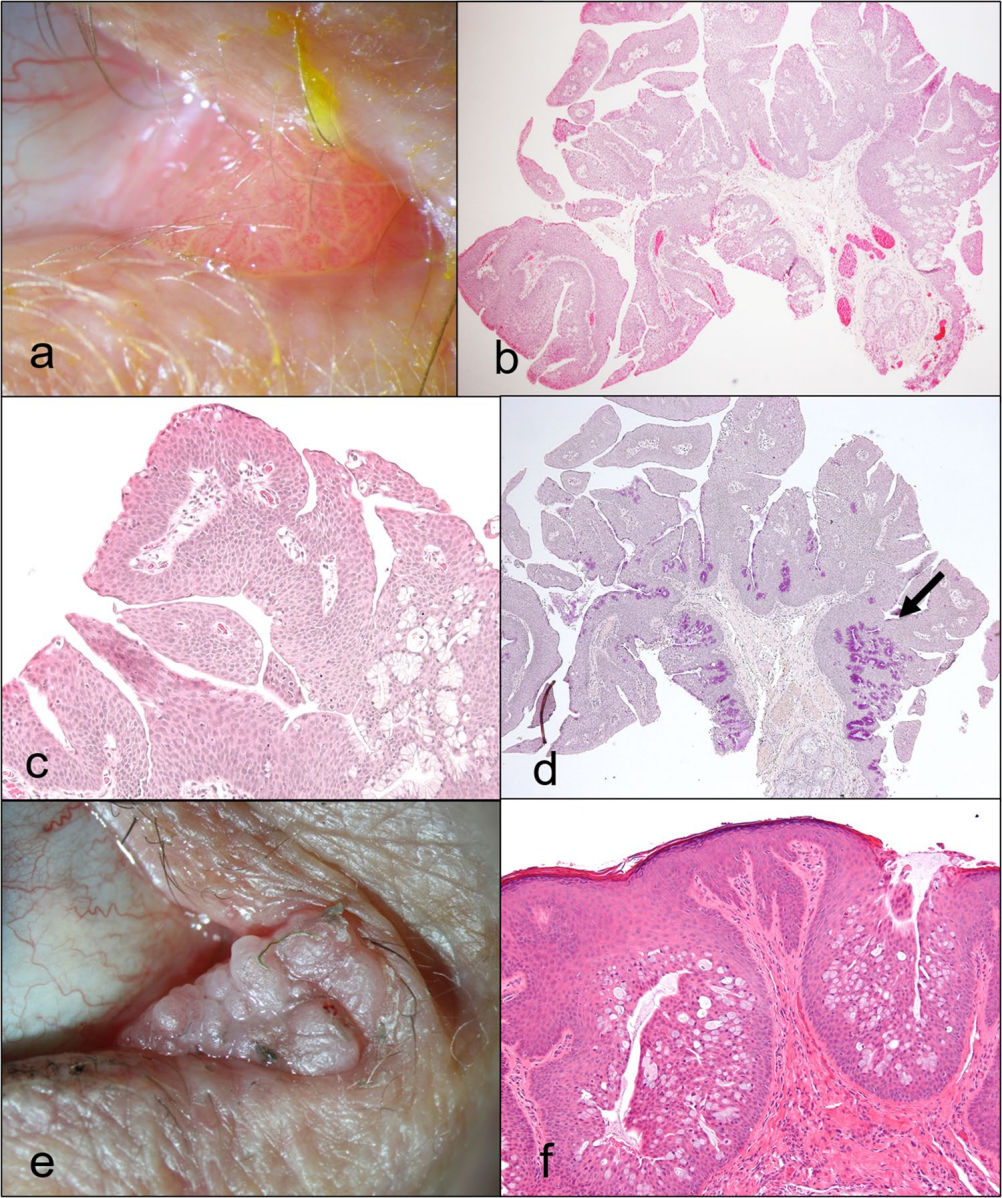
Papilloma. Clinical picture of a reddish, papillomatous lesion of the caruncle (**a**). The corresponding histologic figure shows a hyperplastic, non-keratinized epithelium covering a fibrovascular cord (**b**; H&E stain, 40 ×). Higher magnification shows a non-keratinized thickened epithelium with goblet cells and absence of atypia (**c**; H&E stain, 100 ×). The goblet cells (arrow) are highlighted by a PAS stain (**d**; PAS reaction, 40 ×). Clinical picture of a whitish, papillomatous lesion at the caruncle (**e**). The corresponding histologic figure shows a thickened keratinized squamous epithelium containing many goblet cells (**f**; H&E stain, 100 ×). The keratinization of the epithelium is responsible for the clinical picture of a whitish lesion

Clinical findings: papillomatous epithelial lesion, reddish or whitish.

Histology: proliferating keratinized or non-keratinized acanthotic squamous epithelium with goblet cells covering fibrovascular cords.

#### Sebaceous gland hyperplasia (8.5%, *n* = 7; supplemental Fig. 3)

Clinical findings: yellowish with subepithelial gland-like structures.

Histology: composed of densely packed regular appearing sebaceous glands.

#### Reactive lymphoid hyperplasia (4.9%, *n* = 4; Fig. 4)

**Fig. 4:**
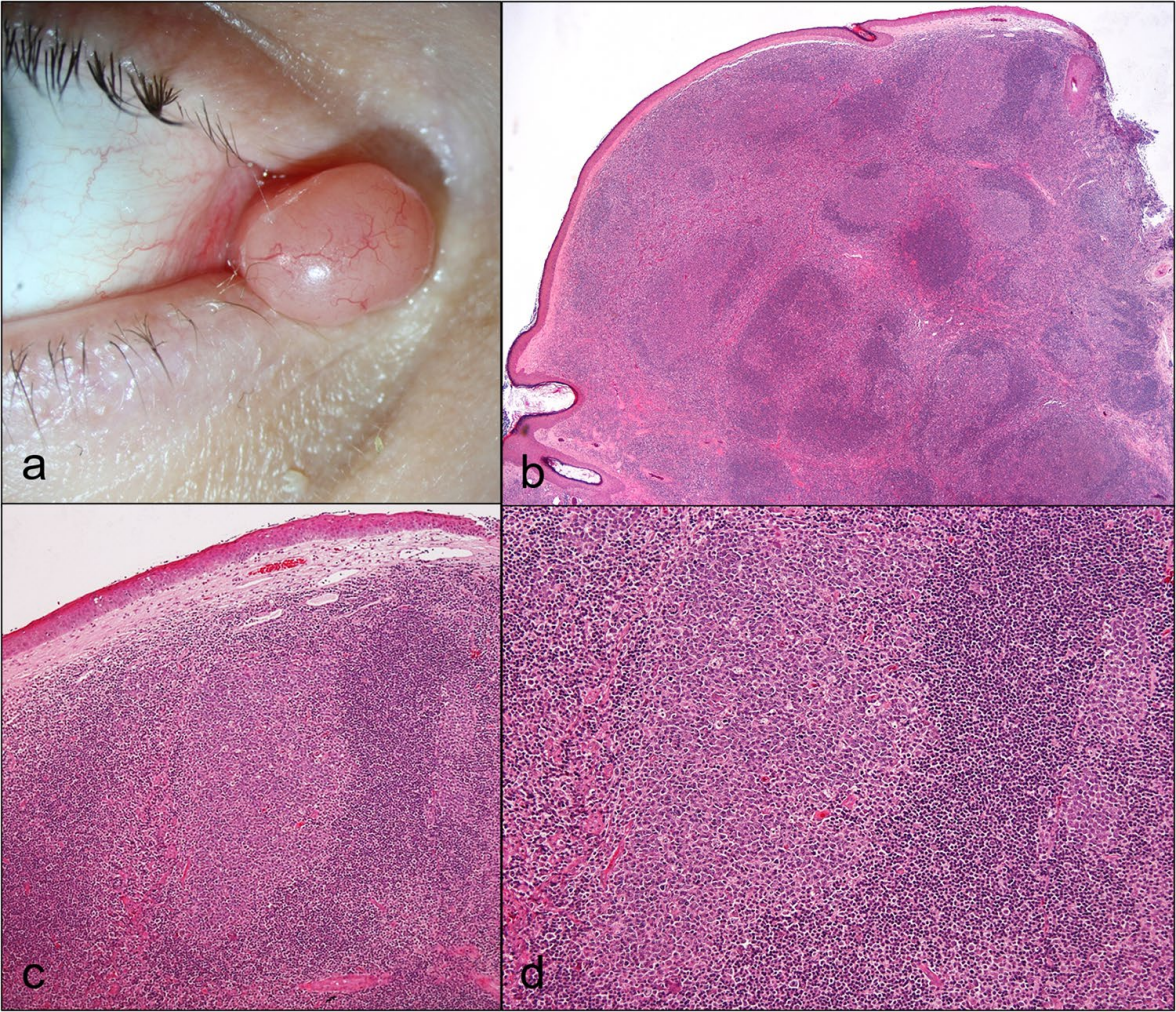
Reactive lymphoid hyperplasia. Clinical pictures of a reddish lesion with a smooth surface (**a**). The corresponding histologic figure shows many lymphocytes arranges in follicles with germinal centers (**b**; H&E stain, 40 ×). Higher magnification of a follicle (**c**; H&E stain, 100 ×) and its germinal center (**d**; H&E stain, 200 ×)

Clinical findings: reddish/salmon-colored, with a smooth surface.

Histology: dense lymphocytic infiltrates with formation of well-defined reactive lymphoid follicles with germinal centers.

#### Others (3.7%, *n* = 3; Fig. 5)

**Fig. 5:**
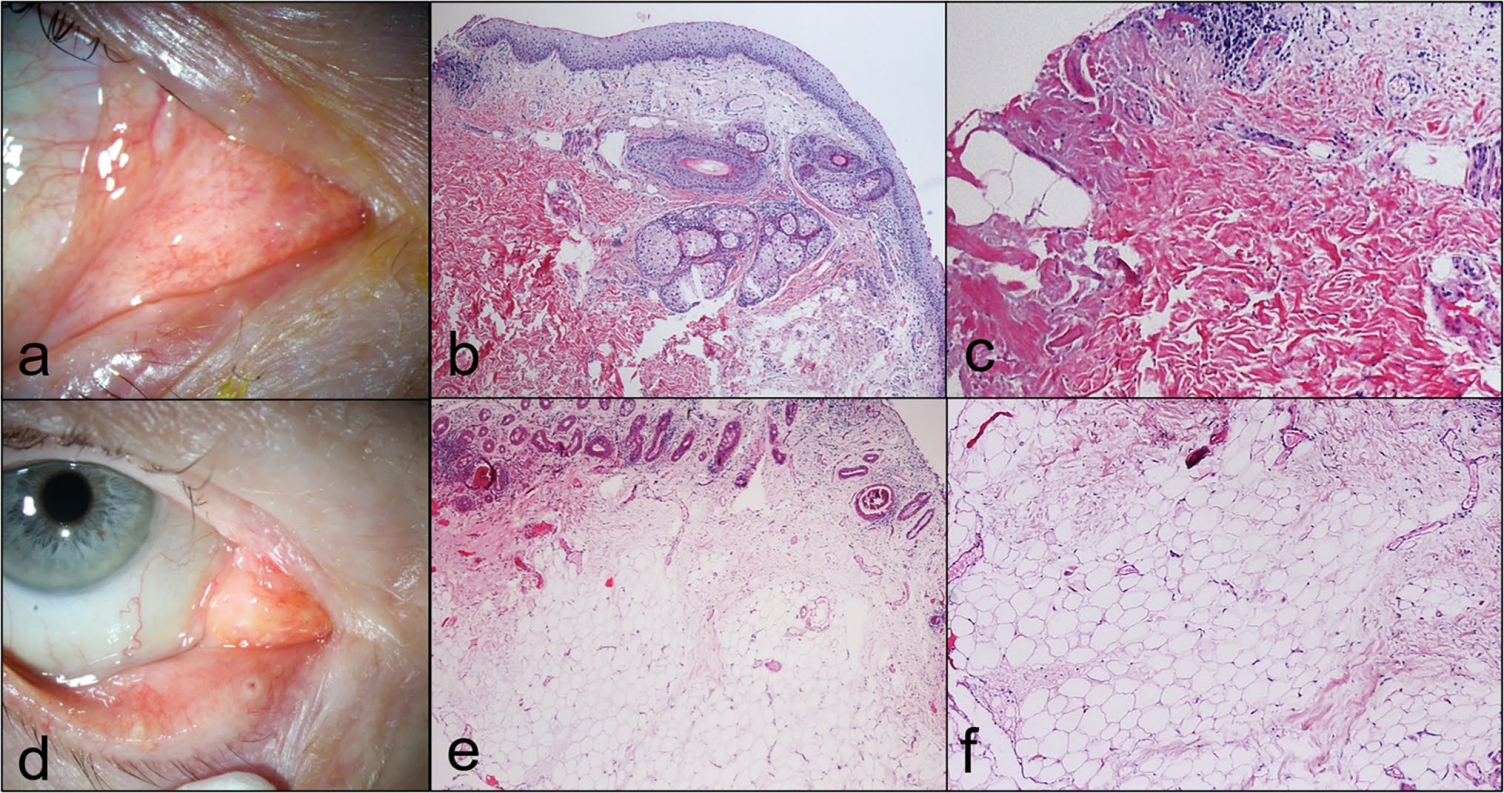
Dermoid. Clinical picture of a reddish to yellowish lesion of the caruncle (**a**). The corresponding histologic figure shows caruncular tissue mainly composed of hair follicles and adjacent sebaceous glands as well as dense collagen lamellae (**b**; H&E stain, 40 ×). Higher magnification illustrates coarse collagen lamellae with occasional fat cells (**c**; H&E stain, 100 ×). Herniated orbital fat. Clinical picture of a yellowish lesion of the caruncle (**d**). The corresponding histologic figure shows predominantly adipose tissue. The reactive lymphoid hyperplasia is not illustrated (**e**; H&E stain, 40 ×). Higher magnification illustrates regular appearing fat cells (**f**; H&E stain, 100 ×)

Normal caruncular tissue; dermoid (thickened collagen lamellae containing skin appendages); herniated orbital fat in the caruncle accompanied by pyogenic granuloma.

### Malignant lesions

#### Conjunctival intraepithelial neoplasia, CIN (1.2%, *n* = 1; for Figure see [12])

Clinical findings: reddish, with adjacent papillomatous surface epithelium.

Histology: conjunctival intraepithelial neoplasia (i.e., intraepithelial proliferation of epithelial cells showing loss of maturation and differentiation) at the base of a typical pyogenic granuloma.

#### Carcinoma (1.2%, *n* = 1; supplemental Fig. 4)

Clinical findings: reddish, smooth surface, with infiltrative growth characteristics.

Histology: subepithelial mass with a lobular growth pattern, cells with prominent nucleoli and a partly foamy cytoplasm, (bizarre) mitotic figures.

## Discussion

The caruncle displays a different spectrum of tumors as the conjunctiva and lesions of the caruncle are rarer [13]. As in our study, the most frequently described caruncular tumors were nevus (24–48%) followed by papilloma (7–32%) [2–11, 14, 15]. There is general agreement that benign caruncular lesions predominate malignant tumors by far (about 94–98%) [1–8, 10, 13, 15, 16]. Only one study of 59 cases reported a higher percentage (15%) of (pre)malignant lesions [5].

Malignant neoplasias at the caruncle comprise basal cell carcinoma, squamous cell carcinoma, basosquamous carcinoma, malignant melanoma, lymphoma, and sebaceous gland carcinoma [5–9, 15, 17–20]. Rare malignant lesions include Kaposi sarcoma [8, 11], rhabdomyosarcoma [21], plasmacytoma [4, 7], metastatic lung carcinoma [22], mucoepidermoid carcinoma [23], and relapse of T cell lymphoma [24].

Although clinicians may aim to make the correct diagnosis clinically (which is more difficult at the caruncle compared to other ocular adnexal sites with a diagnostic accuracy between 37.4 and 87.5% [2, 3, 6, 10]), the most important issue is to reliably identify patients with a (potentially) malignant lesion, e.g., carcinoma, melanoma, CIN, and lymphoid neoplasias (since RLH can be only distinguished from lymphoma histopathologically). In general, as in our study, malignant lesions tend to be overestimated [3, 6]. However, another group reported that they had interpreted two of five malignant caruncular lesion to be benign due to their clinical appearance [3] since malignancies in particular at this site can exhibit an unusual appearance [20] such as melanomas and basal cell carcinoma with the latter exhibiting pigmentation [18].

Based on our own experience and data from the literature, four features should alert the clinician to suspect a carcinomatous malignant process at the caruncle: (1) fast growth, (2) indistinctive borders, (3) smudgy or ulcerated surface, and (4) older age > 60 years (although young age does not exclude malignancy). These criteria do not apply for lymphoid lesions and Kaposi sarcoma [8] which have a different clinical appearance (salmon-colored: lymphoid lesions; reddish: Kaposi sarcoma) can occur at a much younger age. Malignant melanoma has been reported to occur in younger individual as well [2, 7].

Every cohort of caruncular lesions shows a specific spectrum: Our study comprises a relatively high number of oncocytoma comparable to Kaeser [3]. Shields et al. reported a relatively small number of nevi but many pyogenic granulomas which were not found in our study — nor were hemangiomas [1, 3, 7, 15]. RLH was more frequent in the study from Luthra et al. [7] and our study compared to other cohorts. Selected findings of our study are discussed below:

*Nevi* consisted of benign melanocytic lesions (42.7%, *n* = 35) without any evidence of malignant transformation or malignancy. Overall, the mean age was (median: 37.8 years) lower than for the other lesions — except RLH. A slight female preponderance of melanocytic lesions was — in accordance with our study — observed in other studies [3] which may be attributed to hormones in combination with a higher likelihood of self-diagnosis [25]. Eighty-eight percent of the pigmented lesions were clinically correctly identified in contrast to the amelanotic nevi with a correct clinical diagnosis in 30%. None of the patients developed a recurrence although some of the lesions were incompletely excised due to the complex anatomic location. The two combined lesions of papilloma and nevus were clinically diagnosed as papilloma. Junctional activity was more often diagnosed in pigmented compared to amelanotic lesions and typically at a younger age. Focal junctional activity was found up to 57 years with no signs of malignant transformation into a melanoma. In general, conjunctival nevi have a risk of less than 1% for transformation into malignant melanoma [25, 26].

The *cystic lesions* are a heterogeneous group, and therefore the clinical assignment to the final histological diagnosis was difficult since cysts can originate from different structures (e.g., sweat glands, sebaceous glands, surface epithelium). Only 64.3% of the cysts were clinically identified as cystic lesions.

The most frequent cysts diagnosed in our clinic were keratin cysts in which we observed histological differences from epidermoid cysts of the skin. Histologically they were lined by keratinized squamous epithelium, and the lumen contained keratin lamellae. However, a close proximity to sebaceous glands — often opening out into the epithelium of the cyst — were observed in these keratin cysts. Hairs were not present in the lumen of the cyst as one would expect in a dermoid cyst [7] and therefore (and also due to advanced age) could be therefore ruled out as differential diagnosis. However, there is still some confusion regarding dermoid cysts in the literature of caruncular tumors as some authors defined them as normal-appearing skin appendages included in the cyst wall [2]. This definition also applies to our keratin cysts which we, however, do not interpret as genuine dermoid cyst. The dermoid cyst reported by Luthra was removed in a 18-month-old child [7], while the four lesions described by Santos were present in young adults between 18 and 37 years [2] which is comparable to our study. Steatocystoma simplex is a cystic lesion of adulthood (often occurring at the face) lined by stratified squamous epithelium with an eosinophilic cuticle on the inner surface and sebaceous glands communicating with the cystic lumen [27]. This lesion has been previously described at the caruncle in a 23- and a 26-year-old woman [28, 29]. In conclusion, we interpret the presence of sebaceous glands in close proximity to a cystic lumen lined by keratinizing epithelium as a phenomenon which can be attributed to the composition of the caruncle with a high amount of sebaceous glands. We thus prefer the term “keratin cyst” (instead of epidermoid cyst) or “horn cyst” of the caruncle.

*Oncocytoma* is a specific but rare tumor of the caruncle which probably arises from caruncular accessory lacrimal glands named glands of Popoff [30, 31]. It can arise also in the lacrimal gland and other accessory/ectopic lacrimal glands [30, 32].

In our study, patients with oncocytoma were generally older (≥ 55 years; median 70 years) which is in accordance with other studies [1, 6–8, 33, 34]. Only Luthra et al. reported a patient in his thirties with an oncocytoma [7]. Although oncocytoma was already considered in the clinical differential diagnoses in 55.5% (*n* = 5), in only three of these cases it was the main primary clinical diagnosis. Due to their varying clinical appearance (reddish or whitish with a smooth surface and a cystoid, respectively, nodular appearance) they can be mistaken for sebaceous gland hyperplasia, cysts, reactive lymphoid hyperplasia/lymphoma, inflammatory processes, or other kinds of neoplasia, and thus the oncocytomas in this and other series [1, 6] were mainly diagnosed histologically. Two out of four whitish lesions were present for years, three lesions were not recognized by the patient, and the other four lesions (independent of their color) were observed a few months before clinical presentation to our hospital. None of the lesions recurred after surgical removal.

The histologic type of oncocytoma may also affect the clinical appearance as small, solid oncocytomas were more likely to exhibit the classical reddish color (*n* = 5), while larger, cystic oncocytomas were more likely to appear whitish (*n* = 4). However, the transition between these two ends of the spectrum is fluent, and it may be more likely the material in the lumen of the lesion determining the clinical appearance since in particular in larger oncocytomas, surface epithelium (keratinized squamous epithelium or most often non-keratinized epithelium with goblet cells) may become incorporated into the oncocytoma. Different architectural patterns of oncocytoma have been reported before including cystic-micropapillary, confluent-glandular, solid-organized, solid-disorganized [35], respectively, tubular, cystic, solid with tubular or cystic elements [4]. However, there appears to be confusion with these existing classifications [4, 5, 35].

In addition, a pigmented oncocytoma has also been reported before with the pigmentation having been attributed to dense concretions within the tumor [36, 37].

*Papilloma* is one of the lesions (after nevi) at the caruncle which are most frequently surgically excised (7.1–32%) [2, 3, 6–10, 14, 15]. In our cohort, papillomas accounted for 9.8% and were diagnosed in patients from 27 to 67 years with a mean age of 46 years. The clinical picture of a caruncular papilloma is — in accordance with its denomination — a papillomatous lesion which is covered by epithelium and the clinical diagnosis is reliable [2]. The nature of the epithelium determines the clinical appearance, i.e., predominantly keratinized squamous epithelium (= more whitish appearance) or predominantly non-keratinized epithelium (= more reddish appearance). In most papillomas, both keratinized and non-keratinized epitheliums are present in varying amounts. In contrast to the conjunctiva where a papilloma should be covered by non-keratinized epithelium and a loss of goblet cells in combination with keratinization may alert for metaplasia or dysplasia, the caruncle is naturally covered by both types of epithelium, and thus a keratinized epithelial covering can occur regularly in papillomas. However, although not observed in our series, epithelial dysplasia can also (rarely) develop in papillomas of the caruncle as described by Kaeser et al. [3].

*Hyperplasia of sebaceous glands* is a typical lesion of the caruncle. In this cohort, only male patients were affected with an age range from 37 to 83 years. The typical clinical picture is yellowish with a subepithelial gland-like appearance. Thus, in most cases, the correct diagnosis was achieved clinically (83%). The one lesion which had a reddish appearance was clinically diagnosed as pyogenic granuloma. Histopathologic correlation showed a massive inflammatory reaction with numerous vessels around and in parts superior to the sebaceous glands giving the lesion its reddish clinical appearance. The number of sebaceous gland hyperplasia varies between different studies from 1.8 up to 8% (as in our study) [1–3, 6, 7]. However, due to the characteristic clinical appearance, surgical removal is not mandatory explaining the varying frequencies of surgically excised lesions.

*Reactive lymphoid hyperplasia (RLH)* is a rare diagnosis in general. However, it has been shown that RLH is often located in the medial conjunctiva including plica and caruncle [38]. Although RLH can develop nearly at any age, most of the lesions which we have observed at our hospital affect young adults [38]. Of the four lesions at the caruncle in our cohort, the affected patients were also young adults (21, 23, and 34 years old). The lesion of the fourth patient (aged 62) was accompanied by a reactive epithelial hyperplasia. This is in accordance with the literature where most reported patients are younger than 35 years [2, 6, 7]. Patients with caruncular lymphoma were typically older than RLH patients [1–3]. Regarding lymphoma as main differential diagnosis, there is a report of a relapse of a T cell lymphoma manifesting at the caruncle [24].

The clinical diagnosis of RLH at the caruncle is difficult. It may be considered in younger patients and in tumors with a salmon-colored appearance. Once a biopsy is performed showing a lymphoid lesion, it is mandatory to rule out a lymphoma by additional immunohistochemistrical stains and/or clonal analysis since age is not a reliable discriminating factor between RLH and lymphoma.

To the best of our knowledge, we are the first to report herniated orbital fat accompanied by a pyogenic granuloma. Kaeser reported two cases of fatty infiltration of unknown origin in two woman of 51 and 82 years [3]. Since there is no further description, there may be a similarity to our case. Østergaard reported a case of a fibrolipoma which was not further specified [11]. However, in our case, the fat prolapsed during surgery and was partially excised supporting the diagnosis of herniated orbital fat.

Regarding dermoids of the caruncle, one case report of a caruncular dermoid was reported before involving the upper eyelid and palpebral conjunctiva [39]. Østergaard reported a dermoid tumor in a cohort of 574 caruncle tumors [11]. Several other authors use the term “dermoid” synonymous for “dermoid cyst” which may lead to some confusion. A complex choristoma was described in a 3-year-old child [40].

With regard to *malignant epithelial tumors*, we have reported a case series of pyogenic granuloma with a conjunctival intraepithelial neoplasia on its base with one of these lesions occurring at the caruncle [12]. The other patient in our series exhibited a malignant lesion with morphologic features of sebaceous cell carcinoma. However, the immunostains did not unequivocally confirm the diagnosis of sebaceous cell carcinoma, and squamous cell carcinoma has to be considered as differential diagnosis. Together with squamous cell carcinoma, it is one of the most aggressive tumors in this localization since it exhibits an infiltrative growth pattern and can lead to lymph node metastases (which happened to our patient who died despite complete local resection, neck dissection, and radiochemotherapy several months after initial presentation at our hospital).

Although rare, premalignant and malignant epithelial lesions have been reported in nearly every larger cohort on caruncle tumors [1–3, 5–9, 30].

In summary, caruncular tumors have a different spectrum and are less common than lesions of the bulbar and tarsal conjunctiva. They can occur in patients of any age and appear to affect women more often. However, other studies and our cohort only report caruncular lesions that were surgically removed (which is reasonable as the clinical diagnosis may significantly differ from the histological diagnosis) which may explain a higher number of female patients who are more likely to go to a doctor and to have surgery for aesthetic reasons.

Although most caruncular lesions are benign, the clinical diagnosis is often challenging justifying a histopathological examination of each excised lesion to achieve a histological diagnosis and exclude malignant tumors with lethal potential which have in the case of caruncular melanoma a higher risk for metastasis [41].

## Supplementary Information

Supplemental Fig. 1 Diagnosis dependent age distribution of patients with caruncular lesions. (PDF 211 kb)

High resolution image (TIF 4219 kb)

Supplemental Fig. 3 Sebaceous gland hyperplasia. Clinical picture of a yellowish lesion of the caruncle (a). The corresponding histologic figure shows caruncular tissue mainly composed of regularly appearing sebaceous glands (b; H&E stain, 40x). Higher magnification illustrates the holocrinic sebaceous glands (c; H&E stain, 100x) (JPG 659 kb)

High resolution image (TIF 2989 kb)

Supplemental Table Presence of junctional activity in benign melanocytic lesions. (PDF 90 kb)

## Data Availability

Material and data is archived at the Ophthalmic Pathology Laboratory, University of Bonn, Germany.
