# Supplementary figures and images for: Clinico-pathological correlation of lacrimal caruncle tumors: a retrospective analysis over 22 years at the University Eye Hospital Bonn
Source: Graefes Arch Clin Exp Ophthalmol. 2021 Oct 28;260(4):1415–25. doi: 10.1007/s00417-021-05464-x (PMC8913479; doi:10.1007/s00417-021-05464-x)

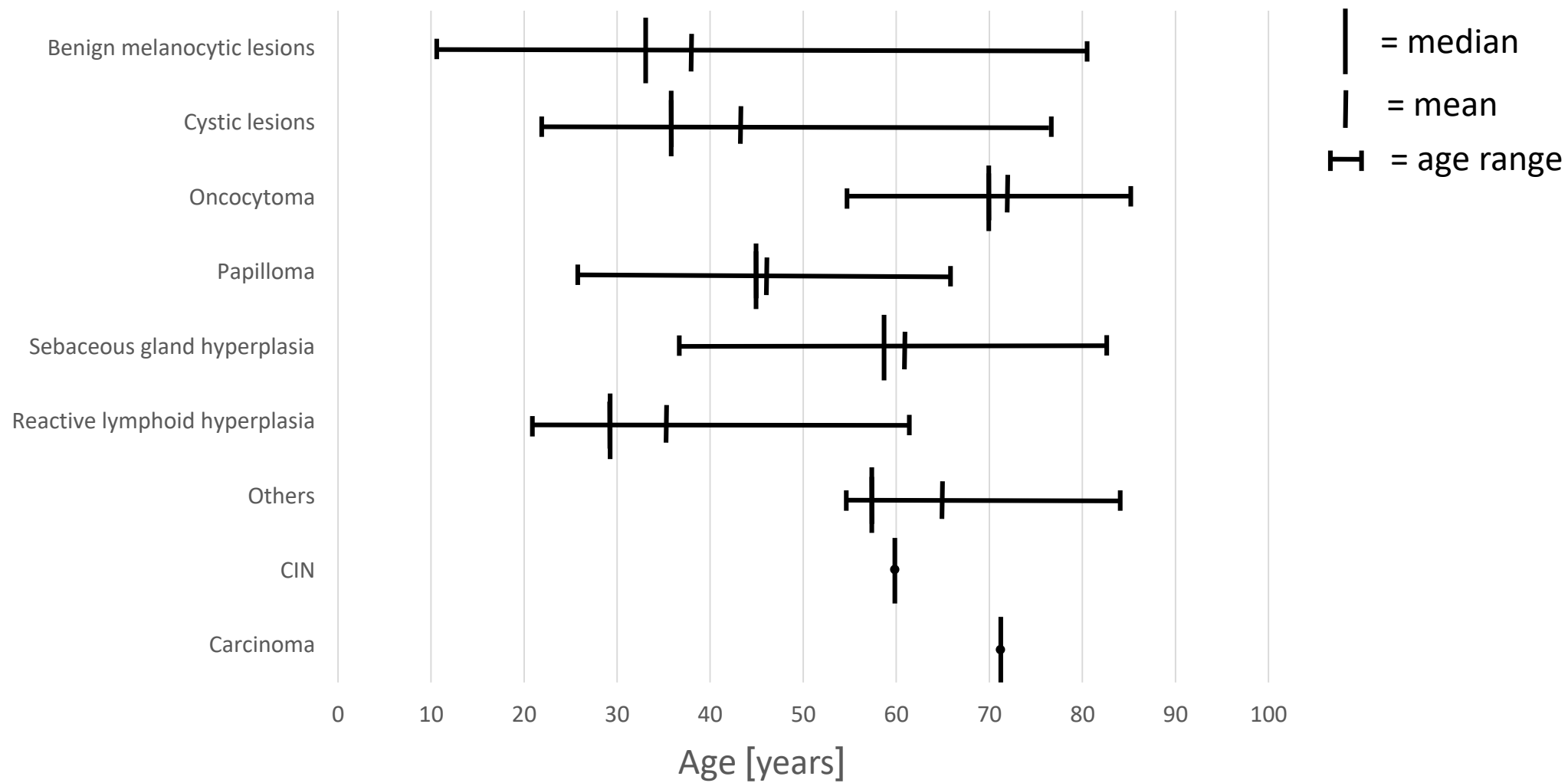

Supplement: Supplementary file 1 — Diagnosis dependent age distribution of patients with caruncular lesions. (PDF 211 kb) [file 417_2021_5464_MOESM1_ESM.pdf]

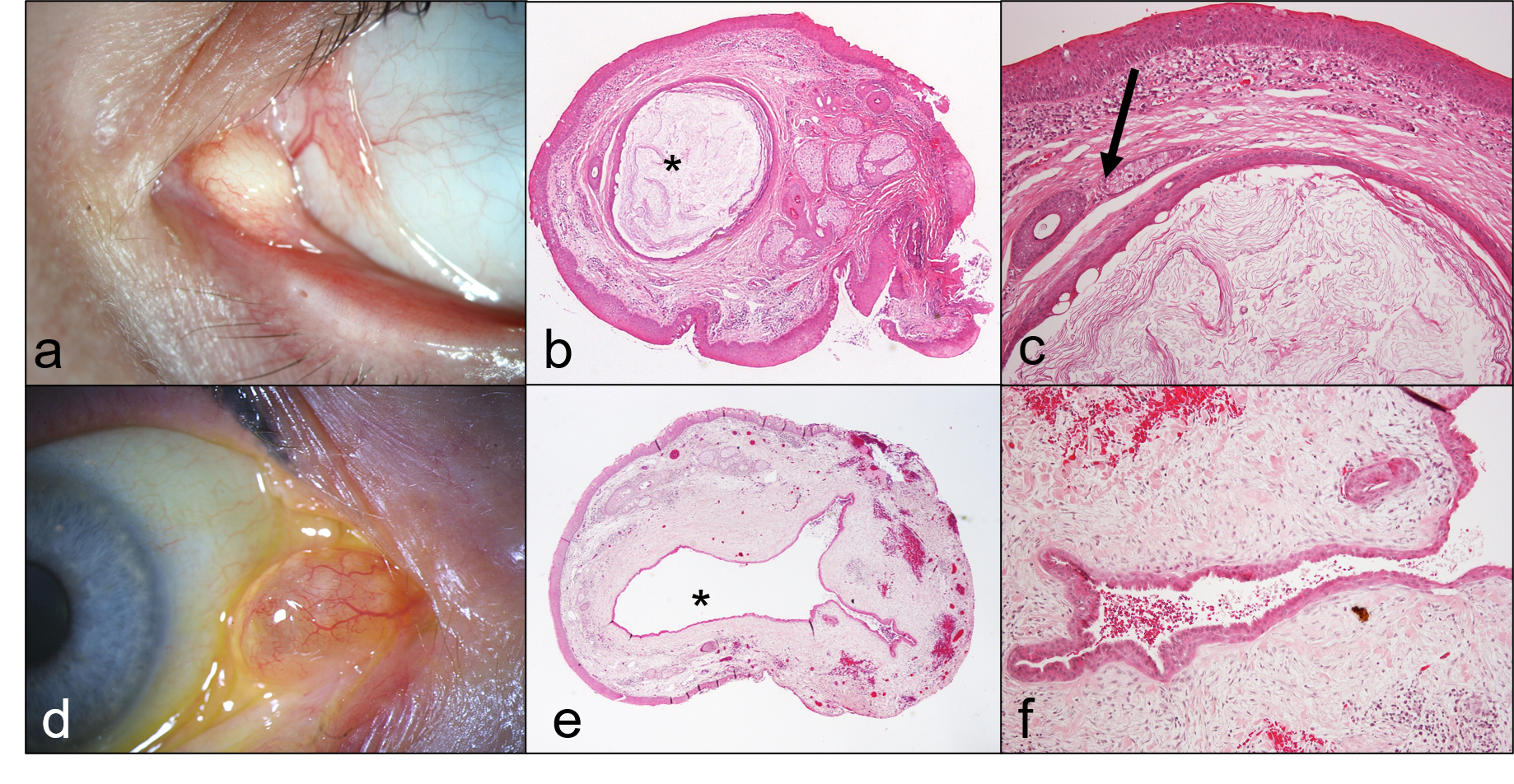

Supplement: Supplementary file 2 — High resolution image (TIF 4219 kb) [file 417_2021_5464_MOESM2_ESM.tif]

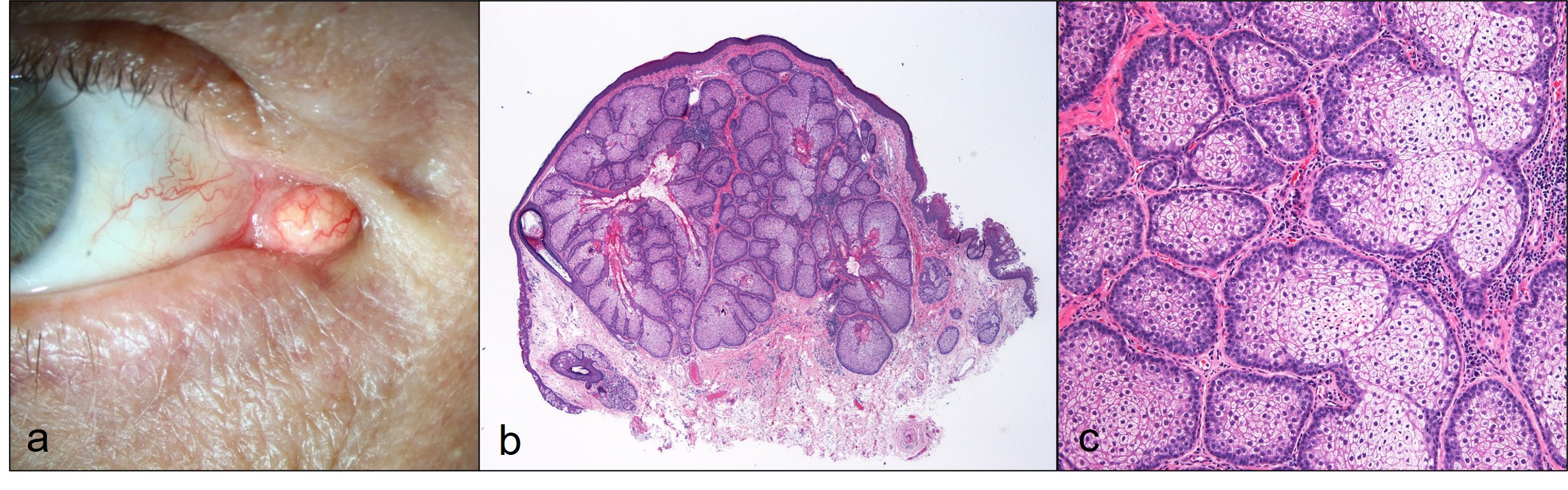

Supplement: Supplementary file 3 — Sebaceous gland hyperplasia. Clinical picture of a yellowish lesion of the caruncle (a). The corresponding histologic figure shows caruncular tissue mainly composed of regularly appearing sebaceous glands (b; H&E stain, 40x). Higher magnification illustrates the holocrinic sebaceous glands (c; H&E stain, 100x) (JPG 659 kb) [file 417_2021_5464_MOESM3_ESM.jpg]

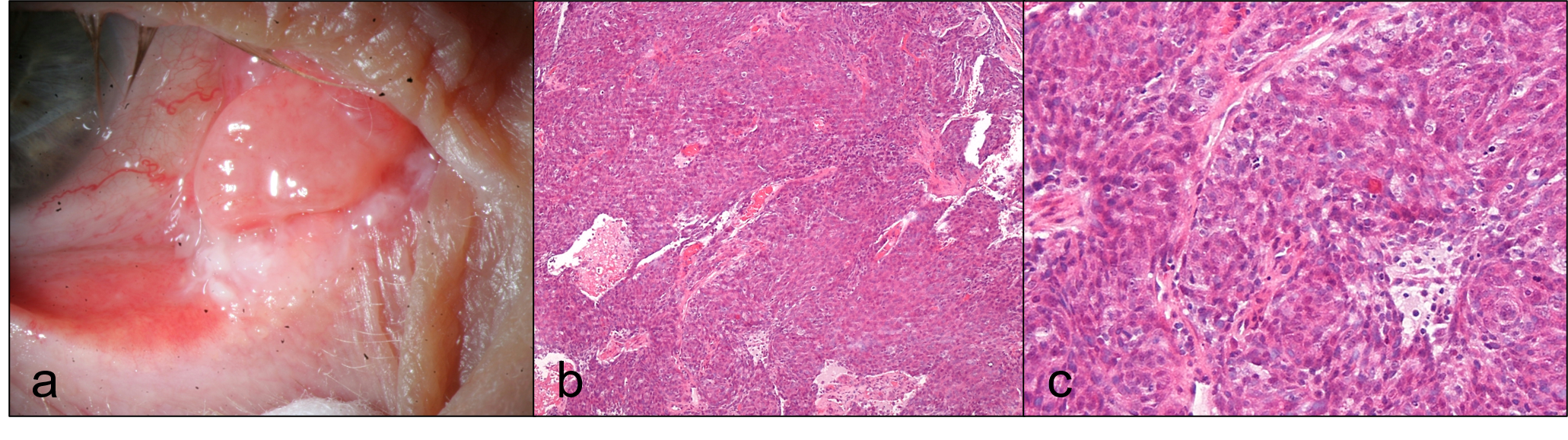

Supplement: Supplementary file 4 — High resolution image (TIF 2989 kb) [file 417_2021_5464_MOESM4_ESM.tif]
