# Supplementary material for: Clinico-pathological correlation of lacrimal caruncle tumors: a retrospective analysis over 22 years at the University Eye Hospital Bonn
Source: Graefes Arch Clin Exp Ophthalmol. 2021 Oct 28;260(4):1415–25. doi: 10.1007/s00417-021-05464-x (PMC8913479; doi:10.1007/s00417-021-05464-x)
Supplement: Supplementary file 5 — Presence of junctional activity in benign melanocytic lesions. (PDF 90 kb) [file 417_2021_5464_MOESM5_ESM.pdf]

Table 2: Presence of junctional activity in benign melanocytic lesions.

**Type of lesion and presence of JA**	**N**	**Age [yrs]**	**Age range [yrs]**
Pigmented lesions	25	38.5	11-70
w/ JA	17	22.6	11-57
w/o JA	8	40.3	26-70
Amelanotic lesions	10	55.2	14-81
w/ JA	2	29.5	14-45
w/o JA	8	61.6.	31-81

Legend: JA=junctional activity; w/=with; w/o=without; yrs=years
